# Left-sided appendicitis in a patient with congenital gastrointestinal malrotation: a case report

**DOI:** 10.1186/1752-1947-1-92

**Published:** 2007-09-19

**Authors:** Frank J Welte, Mario Grosso

**Affiliations:** 1 Department of Radiology, Baystate Medical Center, Tufts University School of Medicine, Springfield, Massachusetts, USA

## Abstract

**Background:**

While appendicitis is the most common abdominal disease requiring surgical intervention seen in the emergency room setting, intestinal malrotation is relatively uncommon. When patients with asymptomatic undiagnosed gastrointestinal malrotation clinically present with abdominal pain, accurate diagnosis and definitive therapy may be delayed, possibly increasing the risk of morbidity and mortality. We present a case where CT was crucial diagnostically and helpful for pre-surgical planning in a patient presenting with an acute abdomen superimposed on complete congenital gastrointestinal malrotation.

**Case presentation:**

A 46-year-old previously healthy male with four days of primarily left-sided abdominal pain, low-grade fevers, nausea and anorexia presented to the Emergency Department. His medical history was significant for poorly controlled diabetes and dyslipidemia. His white blood count at that time was elevated. Initial abdominal plain films suggested small bowel obstruction. A CT scan of the abdomen and pelvis was performed with oral and IV contrast to exclude diverticulitis, revealing acute appendicitis superimposed on congenital intestinal malrotation. Following consultation with the surgical team for surgical planning, the patient went on to laparoscopic appendectomy and did well postoperatively.

**Conclusion:**

Atypical presentations of acute abdominal conditions superimposed on asymptomatic gastrointestinal malrotation can result in delays in delivery of definitive therapy and potentially increase morbidity and mortality if not diagnosed in a timely manner. Appropriate imaging can be helpful in hastening diagnosis and guiding intervention.

## Background

Appendicitis is the most common surgical disease diagnosed in the emergency room setting. Gastrointestinal malrotation is, by comparison, relatively uncommon. Depending upon the location of the cecum and appendix, patients with acute appendicitis and malrotation may present atypically with left-sided abdominal pain. Left-sided abdominal pain most commonly raises the diagnostic question of possible diverticulitis, creating a diagnostic dilemma in these patients. Furthermore, a trend may be developing where diverticulitis, once a disease primarily of older adults, may be becoming more prevalent in younger adults [1] and may exhibit a somewhat different demographic and clinical course [2]. This phenomenon may further bias a clinician confronted with a middle-aged patient with an acute abdomen and left-sided symptoms.

We present a case where the relatively common entity of appendicitis was in no way suspected prior to cross sectional imaging, which incidentally revealed gastrointestinal malrotation, significantly changing clinical management.

## Case presentation

A 46-year-old previously healthy male presented to Emergency Department with four days of primarily left-sided abdominal pain, low-grade fevers, nausea, and anorexia. His medical history was significant for poorly controlled diabetes and dyslipidemia. No history of abdominal surgery was reported. Two months prior to admission, the patient's serum hemoglobin A1C was markedly elevated at 10.7 and his serum lipid profile was quite abnormal (total cholesterol: 313, triglycerides: 287, HDL: 39, LDL: 217). On admission, the patient's home medications included atorvastatin (Lipitor^®^; 40 mg orally per day) and subcutaneous isophane/regular insulin (Insulin Novolin^® ^70/30) twice per day.

The patient's physical exam at presentation was significant for left lower quadrant pain and voluntary guarding. The patient's white blood count at that time was 18.1 × 10^9 ^cells/L (absolute neutrophil count: 13.6; 75.1%), hematocrit: 45.2%, serum glucose: 279, blood urea nitrogen (BUN): 31, and serum creatinine: 1.5. Serum electrolytes were as follows: sodium: 133, potassium: 4.2, chloride: 95, and CO_2_: 23 (anion gap 15). Urinalysis was positive for 2+ albumin, 1+glucose, 1+ ketones, 1+ bilirubin, and hyaline casts. Plain films of the abdomen were obtained in the Emergency Department. A spiral CT scan of the abdomen and pelvis with oral and IV contrast was subsequently performed to exclude colonic diverticulitis.

### Imaging findings

**Figure 1:**
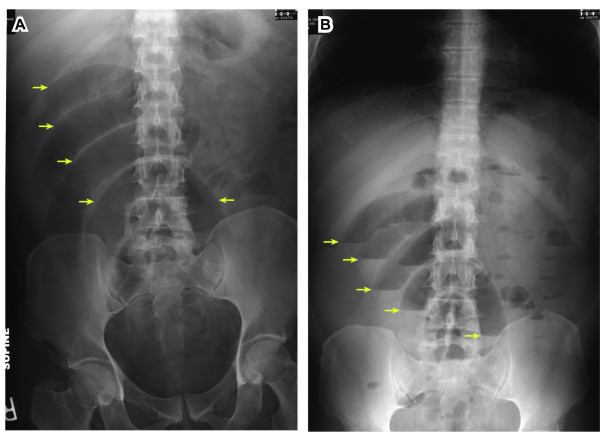
**Abdominal plain films**. Supine (A) and upright (B) abdominal plain films demonstrate multiple loops of dilated small bowel (arrows in A) with air/fluid levels (arrows in B) in the right abdomen, suggestive of small bowel obstruction; this finding can also be seen as an unusual sign of acute appendicitis. Intestinal malrotation was not considered at this time.

Abdominal plain films demonstrated multiple dilated loops of small bowel with air/fluid levels in the right abdomen (arrows in Figure 1), consistent with small bowel obstruction, but also noted, unusually, to herald acute appendicitis. Intestinal malrotation was not considered in the differential diagnosis at that point. No pneumoperitoneum was evident.

**Figure 2:**
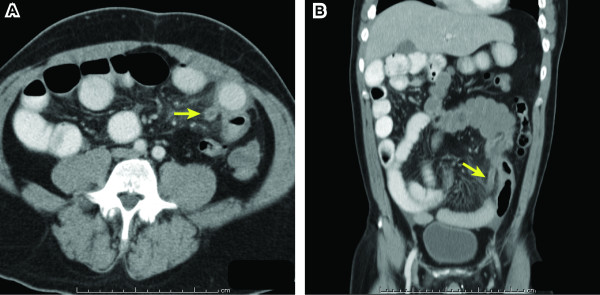
**Abdominal CT: Atypical acute appendicitis**. Axial spiral CT with oral and IV contrast (A) and coronal multiplanar reconstruction (B) demonstrate dilated appendix in the left mid-abdomen (arrows) with adjacent fat stranding.

Abdominal CT with oral and IV contrast was then performed, which demonstrated the majority of the small bowel positioned in the right abdomen, the cecum located in the left mid abdomen, and absence of the ligament of Treitz. The orientation of the superior mesenteric vessels was abnormal. These findings together were consistent with complete intestinal malrotation. No associated situs, caval, or other congenital anomalies were present and no evidence of volvulus was identified. A dilated, tubular, blind-ending structure was identified arising from the cecumin the left mid-abdomen (arrows in Figures 2A,B), with significant stranding in the adjacent fat, indicative of acute appendicitis.

### Management

Fluid resuscitation was initiated in the Emergency Department with Lactated Ringer's solution (125 cc/hr). Based on plain film findings of possible small bowel obstruction and absence of history of any abdominal surgery, an abdominal CT was performed, as discussed above.

After extensive review of the CT data for presurgical planning, the patient was taken to the operating room for laparoscopic exploration and appendectomy, where the imaging findings were confirmed and the appendix was found to be perforated. No surgical intervention to address the malrotation, such as Ladd's procedure, was performed. Pathologic examination of the excised appendix verified the diagnosis of acute gangrenous appendicitis with perforation. The patient's post-operative course was uneventful. The patient was treated perioperatively with a 5 day regimen of intravenous cefoxitin (1,000 mg every 6 hours) and as an outpatient with a 14 day course of oral levofloxacin (500 mg per day; Levaquin^®^).

## Conclusion

Intestinal malrotation represents errors of rotation of the midgut about the superior mesenteric artery during weeks 5–10 of fetal life and subsequent abnormal fixation to the peritoneal wall [3]. Intestinal malrotation, while often associated with other congenital anomalies, is an isolated finding in the majority of adult cases. Approximately 60% of patients with intestinal malrotation present in the first month of life and 20% present between 1 and 12 months, classically with bilious vomiting secondary to duodenal obstruction distal to the Ampulla of Vater. The estimated incidence of intestinal malrotation is 1 in every 500 live births (0.2%; range 0.03 – 0.5%) [3,4] based on autopsy series, retrospective reviews [5], and prospective barium enema studies [6]. The true incidence of adults with asymptomatic malrotation remains difficult to accurately determine.

More typically, symptomatic adults with bowel malrotation present with acute bowel ischemia or bowel obstruction secondary to midgut orcecal volvulus, or with chronic abdominal pain. Treatment of incidentally discovered malrotation in asymptomatic patients older than 1–2 years of age remains somewhat controversial [5,7]. Even in patients in whom a Ladd's procedure is performed, the appendix will be positioned in an abnormal location, unless appendectomy is performed contemporaneously. In the present case, malrotation was incidentally discovered in a previously asymptomatic adult patient presenting with an acute abdomen, confounding the diagnosis.

Other cases of appendicitis in adults or adolescents in the context of intestinal malrotation have been described previously, (see, for example, [4,5,8-15]) frequently with delayed or incorrect initial diagnoses. Several of these prior reports describe diagnosis of acute appendicitis and gastrointestinal malrotation based on abdominal CT, but go on to describe additional imaging such as upper GI or barium enema to verify or further characterize the malrotation prior to surgical intervention. An important point is that if CT is diagnostic of malrotation and of a superimposed acute condition requiring urgent intervention such as acute appendicitis, further immediate dynamic imaging of the intestinal malrotation is often unnecessary, provided the CT is sufficient for surgical planning, as in this case. Furthermore, the sensitivity and specificity of upper GI, fluoroscopic barium enema or abdominal ultrasound in adolescent or adult patients for detection of causes of an acute abdomen are limited compared to CT.

Complete intestinal malrotation can result in common acute clinical entities such as appendicitis presenting atypically due to the *a priori *unexpected location of the cecum and appendix, causing a diagnostic dilemma. In this case, a patient whose clinical presentation was initially most suggestive of diverticulitis was found to actually have acute appendicitis in the context of congenital bowel malrotation.

CT was critical in this case in redirecting the primary clinical team, hastening the administration of definitive therapy, and for presurgical planning. The atypical presentation of acute appendicitis in patients with intestinal malrotation presents a diagnostic challenge. Appropriate imaging can be diagnostically decisive in identifying patients who present with such an atypical presentation of a common emergent clinical entity.

## Competing interests

The author(s) declare that they have no competing interests.

## Authors' contributions

FJW: Primary author; wrote and approved the final manuscript. MG: Provided clinical background and commentary. All authors read and approved the final manuscript.
